# Acute cerebrovascular injury drives chronic reactive heterogeneity of microglia in a mouse model of mixed etiology dementia

**DOI:** 10.1002/alz.095687

**Published:** 2025-01-09

**Authors:** Sophia H Dimas, Jill Roberts, Danielle S. Goulding, Kai Saito, Carlie Cryer, Ben Wendell, Laura Whitnel‐Smith, Josh M. Morganti

**Affiliations:** ^1^ University of Kentucky College of Medicine, Sanders‐Brown Center on Aging, Lexington, KY USA; ^2^ University of Kentucky, Lexington, KY USA; ^3^ Sanders‐Brown Center on Aging, Lexington, KY USA

## Abstract

**Background:**

Vascular contributions to cognitive impairment and dementia (VCID) results from cerebrovascular injuries, significantly contributing to age‐related cognitive decline, and coexists with Alzheimer’s disease (AD) in excess of 70% of AD patients. These co‐occurring neuropathological subtypes are referred to as mixed‐etiology dementia (MED). Despite the prevalence of MED little is known regarding the neuroinflammatory responses of microglia in the context of vascular injury in tissues already containing AD‐related cerebral amyloidosis. In order to begin to understand the susceptibility of microglia in this MED environment we generated a mouse model of MED combining transient stroke in the 5xFAD mouse model of cerebral amyloidosis

**Method:**

Male 5xFAD mice received sham or tandem CCA/MCA unilateral occlusion surgery to induce transient cerebral hypoperfusion model of MED. Post‐surgical intervals spanning sub‐acute (7 days) and chronic (28 days) were examined using histological techniques in tandem with single cell RNA sequencing and spatial transcriptomics.

**Result:**

MED drives progressive cerebral amyloid angiopathy (CAA) accumulation as a function time resulting in a shift of amyloid plaque load from parenchymal domains to significantly higher vascular loads at 28 days. Microglial reactivity is markedly changed due to MED with disruptions in markers associated with homeostatic‐ like reactivity (e.g. P2ry12) towards accumulation of disease‐associated reactivity (e.g. Clec7a). Similarly, we demonstrate that MED drives differential accumulation of vascular injury‐reactive subsets of microglia at both single cell and spatial transcriptomic levels beyond the DAM signatures present in the sham mice and varying as a function of post‐injury interval

**Conclusion:**

MED drives chronic microglial heterogeneity beyond classic DAM signatures, highlighting potential new mechanistic and targetable routes underlying MED neuropathological sequelae

